# ISG15 suppresses ovulation and female fertility by ISGylating ADAMTS1

**DOI:** 10.1186/s13578-023-01024-4

**Published:** 2023-05-11

**Authors:** Yaru Chen, Jiawei Zhou, Shang Wu, Lei Wang, Gaogui Chen, Dake Chen, Xianwen Peng, Yi-Liang Miao, Shuqi Mei, Fenge Li

**Affiliations:** 1 Key Laboratory of Swine Genetics and Breeding of Ministry of Agriculture and Rural Affairs & Key Laboratory of Agricultural Animal Genetics, Breeding and Reproduction of Ministry of Education, Huazhong Agricultural University, Wuhan, 430070 China; 2 Institute of Animal Science and Veterinary Medicine, Hubei Academy of Agricultural Sciences, Wuhan, 430064 China; 3 The Cooperative Innovation Center for Sustainable Pig Production, Wuhan, 430070 China

**Keywords:** Interferon-stimulated gene 15, ISGylation, ADAMTS1, Ovulation, Female fertility, Mice

## Abstract

**Background:**

ISGylation is a post-translational protein modification that regulates many life activities, including immunomodulation, antiviral responses, and embryo implantation. The exact contribution of ISGylation to folliculogenesis remains largely undefined.

**Results:**

Here, *Isg15* knockout in mice causes hyperfertility along with sensitive ovarian responses to gonadotropin, such as increases in cumulus expansion and ovulation rate. Moreover, ISG15 represses the expression of ovulation-related genes in an ISGylation-dependent manner. Mechanistically, ISG15 binds to ADAMTS1 via the ISG15-conjugating system (UBA7, UBE2L6, and HERC6), ISGylating ADAMTS1 at the binding sites Lys309, Lys593, Lys597, and Lys602, resulting in ADAMTS1 degradation via a 20S proteasome-dependent pathway.

**Conclusion:**

Taken together, the present study demonstrates that covalent ISG15 conjugation produces a novel regulatory axis of ISG15-ADAMTS1 that enhances the degradation of ADAMTS1, thereby compromising ovulation and female fertility.

**Supplementary Information:**

The online version contains supplementary material available at 10.1186/s13578-023-01024-4.

## Introduction

Ovarian folliculogenesis in mammals is a remarkably complex, well-orchestrated process in three phases: primordial follicle activation, growth of the follicle, and the ovulatory phase [1–3]. Most follicles undergo growth arrest and atresia, and only a few follicles develop to ovulation [4]. Ovulation is a highly selective process containing a series of events, including meiotic resumption, cumulus expansion, and follicular rupture [5].

As the final gatekeeper in the protein production process, protein post-translational modifications (PTMs), such as phosphorylation, acetylation, and ubiquitination are indispensable for regulating follicle development and ovulation [6]. For instance, ubiquitination and ubiquitin-like modifications, such as SUMOylation and ISGylation, which are involved in oocyte meiotic resumption and primordial follicle reserve [7, 8]. Protein ubiquitination-mediated degradation of protein phosphatase 2A scaffold subunit (PP2A-A) is essential for prophase I arrest and homologous chromosome separation during oocyte meiosis [9]. Moreover, an increase of SUMOylation in SUMO-specific peptidase 1 (*Senp1*) deletion mice impairs early ovarian follicle development by disrupting the stroma-oocyte communication [10]. Thus, further deciphering the detailed pattern of protein ubiquitination or ubiquitin-like modifications in mammalian ovaries will give a clearer understanding of the mechanism underlying follicle development and ovulation are determined.

Interferon (IFN)-stimulated gene 15 (ISG15) is the first reported ubiquitin-like modifier protein [11]. ISG15 contains two ubiquitin-like domains connected by a short linker and a carboxy-terminal LRLRGG motif that is critical for ISG15 conjugation function [12]. ISG15 is covalently conjugated to the substrate lysine residues by a process known as ISGylation [13]. Similar to ubiquitination, ISGylation is catalyzed in a three-step process involving the following enzymes: ubiquitin-like modifier activating enzyme 7 (UBA7) as an ISG15-activating E1 enzyme, ubiquitin-conjugating enzyme E2 L6 (UBE2L6) as an ISG15-conjugating E2 enzyme, and tripartite motif-containing 25 (TRIM25) or hect domain and RLD 6 (HERC6) as an ISG15 E3 ligase [11]. In addition, ISGylation can be reversed by ISG15-specific isopeptidases, such as the ubiquitin carboxy-terminal hydrolase 18 (USP18) [11]. ISG15 has been implicated in several cellular activities, including intracellular transport, immune responses, antiviral defenses, and glucose metabolism [14]. In a mouse model, *Isg15* expression has been shown to dramatically increase in murine endometrium during early pregnancy and *Isg15* depletion impairs implantation and placentation [15, 16], suggesting the important role of *Isg15* in female fertility. However, whether ISG15-mediated protein ISGylation participates in folliculogenesis and female fertility remains unclear.

In this study, we demonstrate that *Isg15* knockout improves reproductive capacity by enhancing the ovulation rate in mice. In line with the role of ISG15 as an ovulation regulator, ISG15 represses the expression of ovulation-related genes in an ISGylation-dependent way. Furthermore, ISG15 ISGylates ADAMTS1 together with the ISG15-conjugating system (UBA7, UBE2L6, HERC6), induces the proteasome-dependent degradation of ADAMTS1 and therefore suppresses the ovulation process. The results of our study reveal a novel role of ISG15 in initiating the ovulatory event. Thus, we have elucidated a critical ISGylation-dependent regulatory mechanism of ovulation and female fertility in mammals.

## Results

### Knockout of Isg15 improves female fertility in mice

**Fig. 1:**
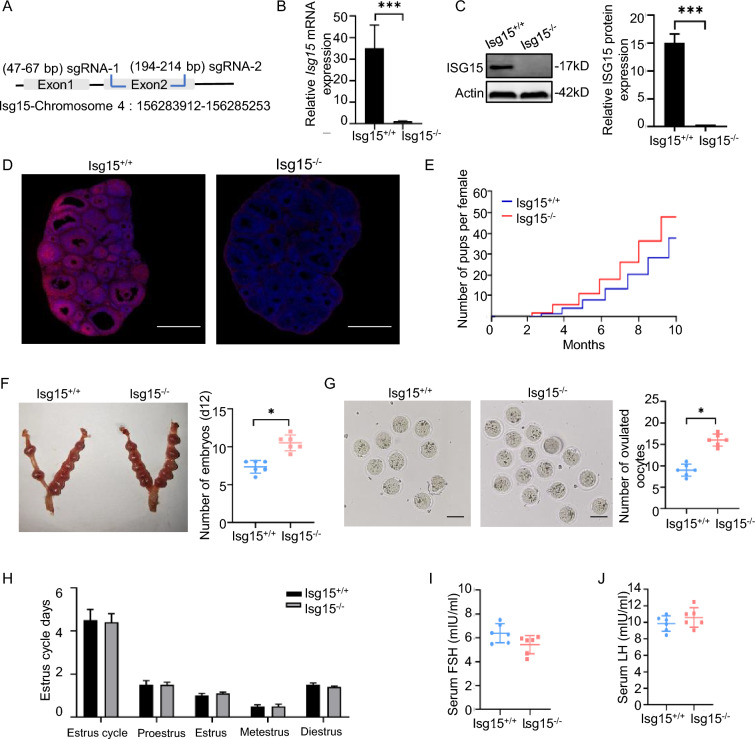
*Isg15* deletion improves female fertility in mice. **A** The schematic diagram of constructing an *Isg15* knockout mouse model. **B** Quantitative RT-PCR analysis of *Isg15* in ovaries of *Isg15*^+*/*+^ and *Isg15*^−/−^ mice.** C** Western blot analysis of ISG15 in ovaries of *Isg15*^+*/*+^ and *Isg15*^−/−^ mice. The right bar graph showed the quantification of protein level. **D** Immunofluorescence analysis of ISG15 in ovaries of *Isg15*^+*/*+^ and *Isg15*^−/−^ mice. Red: ISG15; Blue: DAPI; Scale bars: 500 μm. **E** Cumulating number of pups born per *Isg15*^+*/*+^ (blue line) and *Isg15*^−/−^ (red line) female mice when bred with *Isg15*^+*/*+^ male mice during the eight-month period of fertility test (n = 6). **F** Embryos in *Isg15*^+*/*+^ and *Isg15*^−/−^ mice. The right panel is the number of embryos. **G** Sixteen hours after natural mating, oocytes were collected from *Isg15*^+*/*+^ and *Isg15*^−/−^ oviducts. The rightmost panel is the number of oocytes. Scale bar: 20 μm. **H** Estrus cycle of eight-week-old *Isg15*^+*/*+^ and *Isg15*^−/−^ mice. **I**, **J** Serum FSH (**I**) and LH (**J**) levels in eight-week-old *Isg15*^+*/*+^ and *Isg15*^−/^^−^ mice. All data are mean ± SD; *P < 0.05, ***P < 0.001

To determine the role of ISG15 in fertility in vivo, *Isg15* knockout (*Isg15*^−/−^) mice were generated using CRISPR/Cas9-mediated genome editing (Fig. 1A), followed by confirmation that the *Isg15* gene was not expressed in *Isg15*^−/−^ murine ovaries (Fig. 1B–D). We first investigated the effects of ISG15 on mammalian female fertility and ovarian function. Notably, compared with *Isg15*^+/+^ mice, *Isg15*^−/−^ mice displayed hyperfertility, with increases in litter size during the eight-month breeding period, the number of implanted embryos and the number of naturally ovulated oocytes (Fig. 1E–G). However, *Isg15*^−/−^ mice showed normal estrous cycling, circulating levels of gonadotropins, including follicle-stimulating hormone (FSH) and luteinizing hormone (LH) (Fig. 1H–J).

We then examined male fertility in *Isg15* knockout mice. The *Isg15* gene was not expressed in *Isg15*^−/−^ murine testes (Additional file 1: Fig. S1A–C). *Isg15*^−/−^ male mice were fertile and showed normal serum levels of testosterone (T), FSH, and LH at three and eight weeks of age (Additional file 1: Fig. S1D–F). Meanwhile, three-week-old and eight-week-old *Isg15*^−/−^ male mice shared similarities in testis weight, testis morphology, and epididymis morphology with *Isg15*^+/+^ males (Additional file 1: Fig. S1G–I). And we observed no differences in sperm count and abnormal sperm rate between eight-week-old *Isg15*^+/+^ and *Isg15*^−/−^ male mice (Additional file 1: Fig. S1J, K). Thus, we conclude that *Isg15* depletion causes hyperfertility in females without affecting male fertility.

### Knockout of Isg15 in mice enhances ovulation

**Fig. 2:**
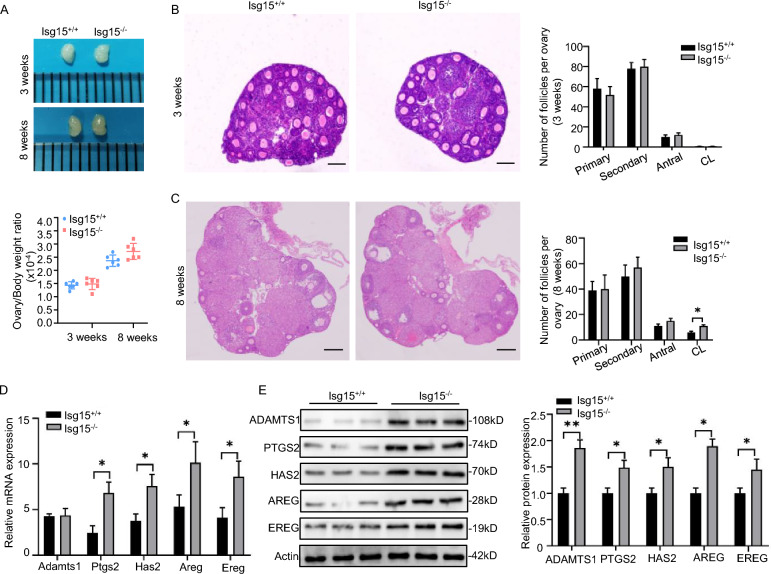
*Isg15* deletion promotes follicular development in mice. **A** The representative ovarian morphology. Ovaries in the representative images were collected from three-week-old and eight-week-old *Isg15*^+*/*+^ and *Isg15*^−/−^ mice. The below is the ratio of ovarian weight to body weight (n = 6). **B**, **C** H&E staining of ovarian sections from three-week-old (**B**) and eight-week-old (**C**) *Isg15*^+*/*+^ and *Isg15*^−/−^ mice. The rightmost panel is the number of follicles at different development stages in three-week-old and eight-week-old mice. CL: corpora lutea. Scale bar: 200 μm. **D**, **E** Quantitative RT-PCR (**D**) analysis and western blot (**E**) analysis of ovulation-related genes including *Adamts1*, *Ptgs2*, *Has2*, *Areg*, *Ereg* in eight-week-old *Isg15*^+*/*+^ and *Isg15*^−/−^ ovaries. The right bar graph showed the quantification of protein level. All data are mean ± SD; *P < 0.05, **P < 0.01

We further assessed the possible impacts of *Isg15* knockout on folliculogenesis. The histology of *Isg15*^−/−^ ovaries at three weeks age was similar to that of the wildtype (Fig. 2A, B); however, by eight weeks of age, *Isg15*^−/−^ ovaries contained more corpora lutea (CL) than the *Isg15*^+/+^ ovaries (Fig. 2C). Indeed, ovulation-related genes, including a disintegrin-like and metalloproteinase with thrombospondin type 1 motif (*Adamts1*), prostaglandin synthase 2 (*Ptgs2*), hyaluronan synthase 2 (*Has2*), amphiregulin (*Areg*), and epiregulin (*Ereg*), were highly expressed in the ovaries of eight-week-old *Isg15*^*−/−*^ mice, compared with *Isg15*^+/+^ mice (Fig. 2D, E).

**Fig. 3:**
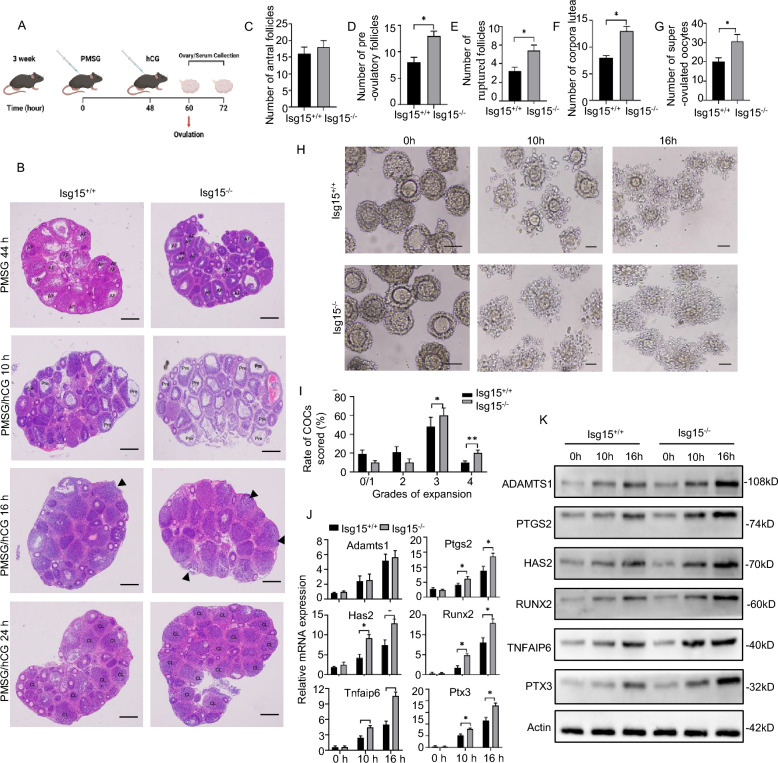
*Isg15* deficiency impacts ovulation in mice. **A** Illustration of the hormonal injection strategy in mice. **B** H&E staining of ovarian sections from three-week-old *Isg15*^+*/*+^ and *Isg15*^−/−^ mice stimulated by PMSG/hCG. AF: antral follicle. Pre: pre-ovulatory follicle. CL: corpora lutea. Black triangles show ruptured follicles. Scale bar: 200 μm. **C** Forty-four hours after PMSG injection, the number of antral follicles was counted in *Isg15*^+*/*+^ and *Isg15*^−/−^ ovaries. **D** Ten hours after PMSG/hCG injection, the number of pre-ovulatory follicles was counted in *Isg15*^+*/*+^ and *Isg15*^−/−^ ovaries. **E** Sixteen hours after PMSG/hCG injection, the number of ruptured follicles was counted in *Isg15*^+*/*+^ and *Isg15*^−/−^ ovaries. **F** Twenty-four hours after PMSG/hCG injection, the number of corpora lutea was counted in *Isg15*^+*/*+^ and *Isg15*^−/−^ ovaries. **G** The number of oocytes ovulated by *Isg15*^+*/*+^ and *Isg15*^−/−^ mice after superovulation treatment (n = 6). **H** Cumulus-oocyte complexes (COCs) isolated from *Isg15*^+*/*+^ and *Isg15*^−/−^ mice were cultured in vitro for 0 h, 10 h, 16 h. Scale bar: 20 μm. **I** Cumulus expansion of COCs from *Isg15*^+*/*+^ and *Isg15*^−/−^ mice was scored after 16 h in vitro culture (n = 50 for each group). **J, K** Expression of cumulus expansion-linked genes including *Adamts1*, *Ptgs2*, *Has2*, *Runx2*, *Tnfaip6*, *Ptx3* were assessed by quantitative RT-PCR (**J**) and western blot (**K**) in the cumulus cells from *Isg15*^+*/*+^ and *Isg15*^−/^^−^ mice. All data are mean ± SD; *P < 0.05

To further confirm whether the hyperfertility in *Isg15*^−/−^ females was attributed to a high ovulation rate, we observed ovulation in vivo using pregnant mare serum gonadotropin/human chorionic gonadotropin (PMSG/hCG) primed immature mice (Fig. 3A) [17]. We then used H&E staining to analyze the ovarian histology during ovulation. The results revealed that the number of antral follicles was similar between the *Isg15*^−/−^ and *Isg15*^+/+^ mice treated with PMSG (Fig. 3B, C). There were more pre-ovulatory follicles, ruptured follicles, and corpora lutea in *Isg15*^*−/−*^ ovaries than in normal ovaries treated with PMSG and hCG (Fig. 3D–F), suggesting that *Isg15* deficiency could facilitate the transition from antral follicular to the mature stage. *Isg15* depletion led to marked increases in the number of super-ovulated oocytes (Fig. 3G). Together, these results suggest that *Isg15*^−/−^ mice are much more sensitive to gonadotropin stimulation than are *Isg15*^+/+^ mice. The cumulus expansion of cumulus-oocyte complexes (COCs) was then checked as it is critical to the LH-induced ovulation [18]. The results showed *Isg15* deletion promoted cumulus expansion (Fig. 3H, I) and elevated the expression of cumulus expansion-linked genes, including *Adamts1*, *Ptgs2*, *Has2*, runt-related transcription factor 2 (*Runx2*), tumor necrosis factor alpha-induced protein 6 (*Tnfaip6*), and pantraxin-3 (*Ptx3*) (Fig. 3J, K and Additional file 2: Fig. S2A–C). Based on these results, we conclude that *Isg15* deletion promotes cumulus expansion, which contributes to enhance ovulation.

### ISG15 regulates the expression of ovulation-related genes in an ISGylation-dependent way in murine granulosa cells

**Fig. 4:**
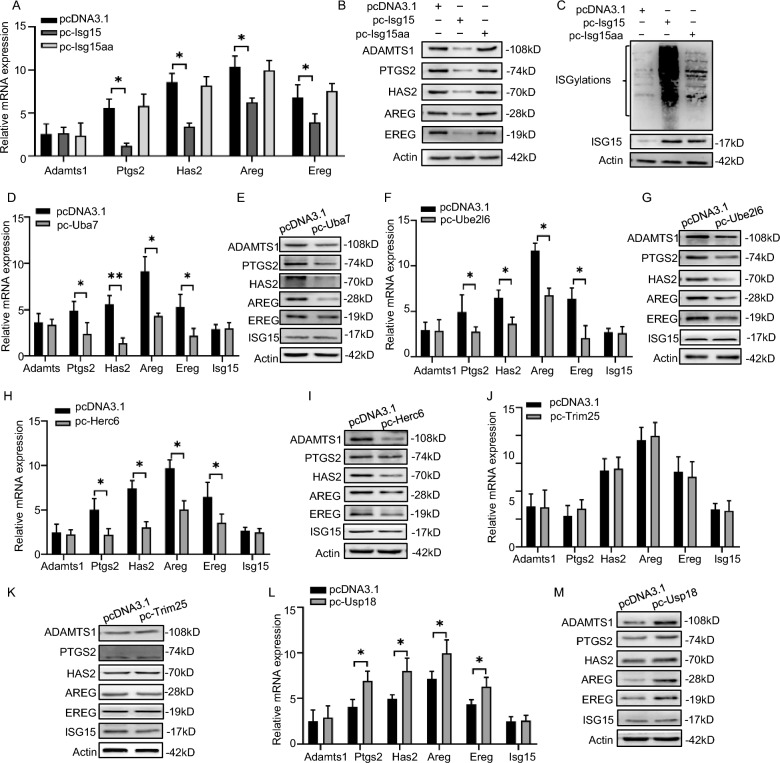
ISG15 negatively regulates ovulation-related genes expression in an ISGylation-dependent way in mGCs. **A**, **B** Quantitative RT-PCR (**A**) analysis and western blot (**B**) analysis of ovulation-related genes including *Adamts1*, *Ptgs2*, *Has2*, *Areg*, *Ereg* in mGCs overexpressing *Isg15* and *Isg15aa*. mGCs were transfected with pcDNA3.1, pcDNA3.1-*Isg15* or pcDNA3.1-*Isg15aa* (ISG15 conjugation-defective mutant) for 48 h and cell lysates were collected for assays. **C** ISGylation level in mGCs overexpressing *Isg15* and *Isg15aa*. mGCs were transfected with pcDNA3.1, pcDNA3.1-*Isg15* or pcDNA3.1-*Isg15aa* for 48 h and cell lysates were collected for assays. **D**, **E** Quantitative RT-PCR (**D**) analysis and western blot (**E**) analysis of ovulation-related genes including *Adamts1*, *Ptgs2*, *Has2*, *Areg*, *Ereg*, *Isg15* in mGCs overexpressing E1 activating enzyme *Uba7*. **F**, **G** Quantitative RT-PCR (**F**) analysis and western blot (**G**) analysis of ovulation-related genes including *Adamts1*, *Ptgs2*, *Has2*, *Areg*, *Ereg*, *Isg15* in mGCs overexpressing E2 conjugating enzyme *Ube2l6*. **H**, **I** Quantitative RT-PCR (**H**) analysis and western blot (**I**) analysis of ovulation-related genes including *Adamts1*, *Ptgs2*, *Has2*, *Areg*, *Ereg*, *Isg15* in mGCs overexpressing E3 ligating enzyme *Herc6*. **J**, **K** Quantitative RT-PCR (**J**) analysis and western blot (**K**) analysis of ovulation-related genes including *Adamts1*, *Ptgs2*, *Has2*, *Areg*, *Ereg*, *Isg15* in mGCs overexpressing E3 ligating enzyme *Trim25.* **L**, **M** Quantitative RT-PCR (**L**) analysis and western blot (**M**) analysis of ovulation-related genes including *Adamts1*, *Ptgs2*, *Has2*, *Areg*, *Ereg*, *Isg15* in mGCs overexpressing ISGylation-specific deconjugating enzyme *Usp18*. All data are mean ± SD; *P < 0.05, **P < 0.01

Subsequently, we used functional loss and gain to assess the effects of *Isg15* in murine granulosa cells (mGCs). We found *Isg15* suppressed the expression of ovulation-related genes, including *Adamts1*, *Ptgs2*, *Has2*, *Areg*, and *Ereg* in mGCs (Fig. 4A, B and Additional file 3: Fig. S3A, B). Because ISG15 is best known for its covalent conjugation to target proteins (ISGylation) [19], we then checked the level of ISGylation in the ovarian tissues. The results demonstrated that the ISGylation level was reduced in hyper-fertile *Isg15*^−/−^ ovaries compared with *Isg15*^+*/*+^ ovaries (Additional file 3: Fig. S3C). An ISGylation-inactivated mutant (*Isg15aa)* with the C-terminal LRLRGG mutated to LRLRAA was constructed and expressed in mGCs (Fig. 4C). Notably, *Isg15aa* overexpression hardly affected the expression of ovulation-related genes (Fig. 4A, B). Furthermore, the ISGylation-specific E1 (*Uba7*), E2 (*Ube2l6*), and E3 (*Herc6*) enzymes, but not the E3 (*Trim25*) enzyme, suppressed the expression of ovulation-related genes (Fig. 4D–K and Additional file 3: Fig. S3D–K). Conversely, the deISGylation enzyme* Usp18* promoted the expression of ovulation-related genes (Fig. 4L, M and Additional file 3: Fig. S3L, M). In conclusion, ISG15 inhibits ovulation-related genes expression in an ISGylation-dependent manner in mGCs.

### ADAMTS1 is a target protein for ISGylation

**Fig. 5:**
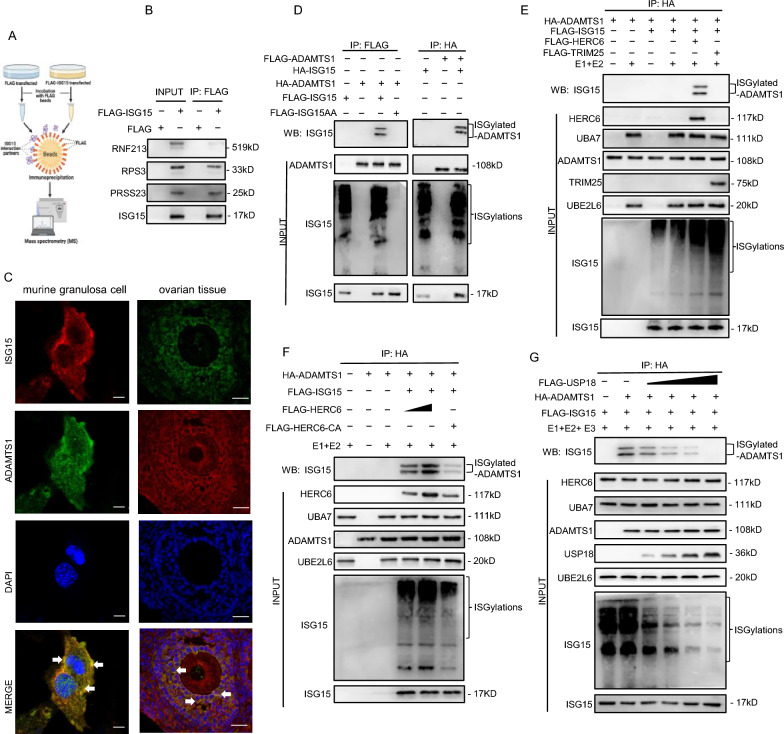
ADAMTS1 is covalently modified by ISG15 in mGCs. **A** Workflow of immunoprecipitation-mass spectrometry. **B** Immunoprecipitation assays between ISG15 and ISG15-binding candidates (RNF213, RPS3, PRSS23). mGCs were transfected with FLAG-ISG15 and cell lysates were immunoprecipitated with anti-FLAG beads. **C** Colocalization analysis of endogenous ISG15 with ADAMTS1 by immunofluorescence assays in mGCs (left panel) and ovaries (right panel). Left panel: Anti-ISG15 and CY3-conjugated goat anti-rabbit IgG (H + L) antibodies were used to detect ISG15 (red); Anti-ADAMTS1 and FITC-conjugated goat anti-rabbit IgG (H + L) antibodies were used to detect ADAMTS1 (green); The nuclei were stained by DAPI (blue); Scale bar: 10 μm. Right panel: Anti-ISG15 and FITC-conjugated goat anti-rabbit IgG (H + L) antibodies were used to detect ISG15 (green); Anti-ADAMTS1 and CY3-conjugated goat anti-rabbit IgG (H + L) antibodies were used to detect ADAMTS1 (red); The nuclei were stained by DAPI (blue); Scale bar: 20 μm. **D** ADAMTS1 is a novel interaction protein of ISG15 using immunoprecipitation assays. mGCs were transfected with HA-ADAMTS1, FLAG-ISG15 or FLAG-ISG15-AA (ISG15 conjugation-defective), HA-ISG15, FLAG-ADAMTS1. Cell lysates were subjected to immunoprecipitation assays using anti-HA or anti-FLAG beads. **E** HERC6 is an E3 ligase for ADAMTS1 ISGylation. mGCs were transfected with HA-ADAMTS1, FLAG-ISG15, FLAG-UBA7, FLAG-UBE2L6, FLAG-HERC6 or FLAG-TRIM25 and then cell lysates were immunoprecipitated with anti-HA beads. **F** ISGylation of ADAMTS1 depends on the activity of E3 ligase HERC6. mGCs were transfected with HA-ADAMTS1, FLAG-ISG15, FLAG-HERC6-WT or FLAG-HERC6-CA (HERC6 ligase-deficient mutant). Cell lysates were immunoprecipitated with anti-HA beads. **G** USP18 decreases ADAMTS1 ISGylation. HA-ADAMTS1, FLAG-ISG15, FLAG-UBA7, FLAG-UBE2L6, FLAG-HERC6 were transfected into mGCs, together with 3 μg, 6 μg, 9 μg, or 12 μg of FLAG-USP18 plasmid. Cell lysates were immunoprecipitated with anti-HA beads

To identify the target proteins for ISGylation in ovulation, we performed immunoprecipitation-mass spectrum (IP-MS) analysis to identify potential ISG15-interacting proteins in mGCs. Briefly, we immunoprecipitated ISG15 from mGCs expressing FLAG-ISG15 and then identified these immunoprecipitates through mass spectrometry (MS) (Fig. 5A). ISG15-binding candidates included ring finger protein 213 (RNF213), which is known as an ISG15 interactor and sensor of ISGylated substrates [20], indicating that the MS data is credible (Additional file 4: Table S1). We further validated the interaction between ISG15 and ISG15-binding candidates (RNF213, RPS3, and PRSS23) by immunoprecipitation assays (Fig. 5B). Notably, ADAMTS1, a prerequisite for successful ovulation, appeared in the ISG15-binding candidate list for the first time. The colocalization of endogenous ISG15 and ADAMTS1 in the cytoplasm (Fig. 5C), detected by confocal analysis in murine granulosa cells and ovaries, indicates the possibility that ISG15 interacts with ADAMTS1. Moreover, immunoprecipitation assays revealed that ADAMTS1 was modified by ISG15 in *Isg15* overexpressed mGCs but not in *Isg15aa* mGCs, which suggested that ADAMTS1 is covalently conjugated to ISG15 (Fig. 5D). The E3 ligases are responsible for recognizing specific substrates and catalyzing their ISGylation. The results of immunoprecipitation assays also demonstrated that HERC6 but not TRIM25 efficiently modified the ISGylation of ADAMTS1 as the E3 ligase (Fig. 5E). In addition, HERC6 could regulate the ISGylation level of ADAMTS1 in mGCs, whereas catalytically inactive mutants (HERC6-CA) could not, indicating that the catalytic activity of E3 ubiquitin ligase is critical for ADAMTS1 ISGylation (Fig. 5F). Consistently, USP18 was able to efficiently reverse the ISG15 modification from ADAMTS1 in a dose-dependent manner (Fig. 5G). Taken together, these results suggest that ADAMTS1 can be ISGylated by ISG15.

### Lys309, Lys593, Lys597, and Lys602 are the ISGylation sites on ADAMTS1

**Fig. 6:**
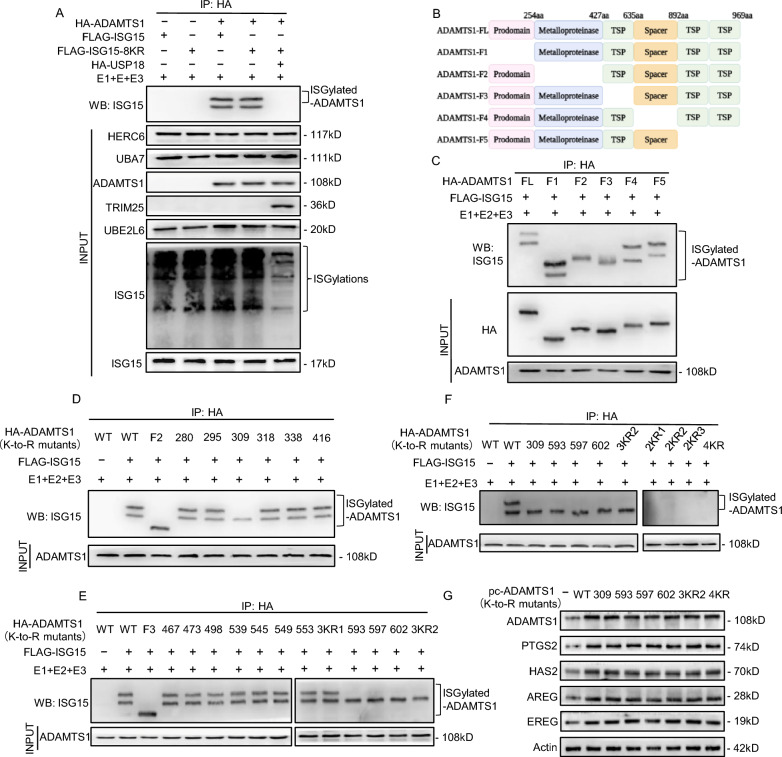
Lys309, Lys593, Lys597, and Lys602 are the covalent ISG15 conjugation sites on ADAMTS1. **A** ADAMTS1 contains at least 2 ISGylation sites. mGCs were transfected with HA-ADAMTS1, HA-USP18, FLAG-ISG15 or FLAG-ISG15-8KR (ISG15 Lys-less variant). Cell lysates were immunoprecipitated with anti-HA beads. **B** Schematic representation of full-length ADAMTS1 (FL) and its deletion mutants (F1-F5). TSP: thrombospondin type 1 repeats. **C** Mapping the interacting regions in ADAMTS1 with ISG15. mGCs were transfected with FLAG-ISG15, HA-ADAMTS1, the truncation mutants of ADAMTS1 (F1-F5). Cell lysates were immunoprecipitated with anti-HA beads. **D** Identification of ISGylation sites on ADAMTS1 F2. **E** Identification of ISGylation sites on ADAMTS1 F3. 3KR1: K545/549/553R. 3KR2: K593/597/602R. **F** Lys309, Lys593, Lys597, and Lys602 are ISGylation sites on ADAMTS1. 2KR1: K309/593R, 2KR2: K309/597R, 2KR3: K309/602R, 4KR: K309/593/597/602R. (**D**-**F**) HA-ADAMTS1 is subjected to mutagenesis for substituting the Lys residues with Arg residues. mGCs were transfected FLAG-ISG15, HA-ADAMTS1 and the indicated point mutants of ADAMTS1 (K-to-R mutants). Cell lysates were immunoprecipitated with anti-HA beads.** G** Western blot analysis of ovulation-related genes including *Adamts1*, *Ptgs2*, *Has2*, *Areg*, *Ereg* in mGCs overexpressing ADAMTS1 mutants

Normally, ISG15 molecules can be conjugated to lysine residues on target proteins [12]. As two ISGylated ADAMTS1 bands appeared in mGCs overexpressing *Isg15* and ISG15-conjugating system (E1, E2, E3) (Fig. 5D–G), we hypothesized that ADAMTS1 might contain at least two ISGylation sites. To rule out the possibility that a dimeric ISG15 chain might be ligated to a single site, an ISG15 variant (ISG15-8KR) was generated by replacing all eight Lys residues on ISG15 with Arg residues. ISG15-8KR also led to the appearance of doubly ISGylated ADAMTS1, which could be eliminated in *Usp18* co-expressed mGCs (Fig. 6A), implying that there might be at least two Lys residues on ADAMTS1.

To determine the targeting sites of ISGylation on ADAMTS1, five truncation mutants of ADAMTS1 were constructed (Fig. 6B). Notably, the upper ISGylation band, which might denote the conjugation of two ISG15 molecules [12, 21, 22], was effectively abolished with the deletion of amino acids 254–426 (F2) or amino acids 427–634 (F3), supporting that ISGylation sites are located within amino acids 254 to 634 (Fig. 6C). We next attempted to determine the Lys (K) residues on ADAMTS1 that are subject to ISGylation. ADAMTS1 mutants in which Lys309, Lys593, Lys597, or Lys602 were separately replaced with Arg (K309R, K593R, K597R, or K602R) resulted in the appearance of the lower ISGylation band that represents the conjugation of a single ISG15 molecule (Fig. 6D, E). Because the neighboring Lys residues might provide alternative sites for ISGylation, we generated a triple mutant 3KR2 (K593/597/602R), which was also found to produce the lower ISGylation band (Fig. 6E). Moreover, we further constructed three double mutants 2KR1 (K309/593R), 2KR2 (K309/597R), and 2KR3 (K309/602R), which led to the disappearance of both the upper and lower ISGylation bands (Fig. 6F). These results suggested that ADAMTS1 could be equally modified at any one of the ISGlyation sites (Lys593, Lys597, Lys602). The quadruple mutant 4KR (K309/593/597/602R) led to the disappearance of both the upper and lower ISGylation bands (Fig. 6F), which indicated that Lys309 and one of the three lysines (Lys593, Lys597, Lys602) are constitutive sites of ADAMTS1 ISGylation. It is noteworthy that the mutation of ISGylation sites did not affect *Adamts1* expression and function (Fig. 6G). Thus, we conclude that Lys309, Lys593, Lys597, and Lys602 are the alternative ISGylation sites on ADAMTS1.

### ISGylation induces ADAMTS1 degradation in a proteasome-dependent manner

**Fig. 7:**
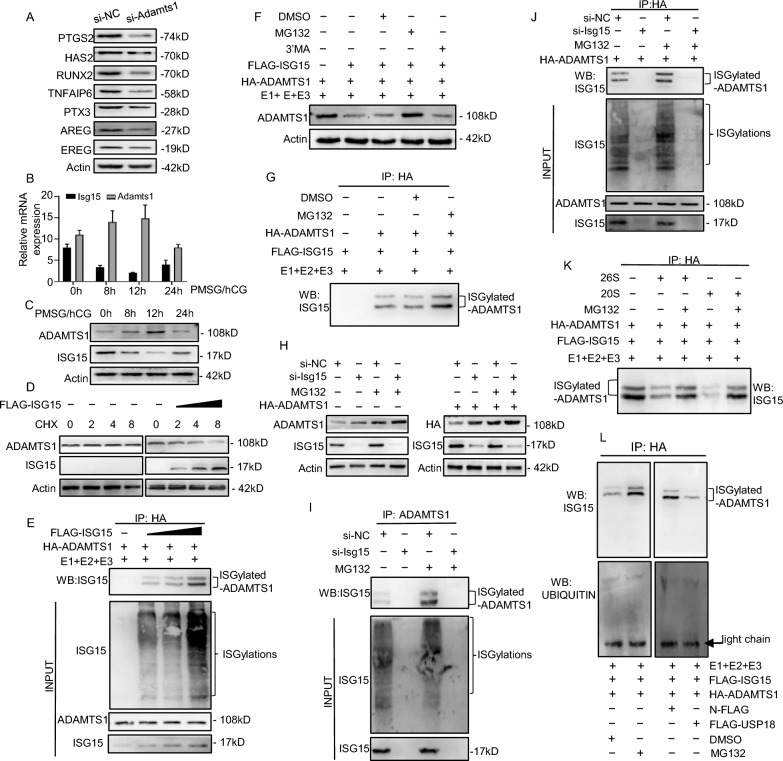
ADAMTS1 degradation is mediated by ISGylation-proteasome pathway. **A** Western blot analysis of cumulus expansion-linked and ovulation-related genes including *Ptgs2*, *Has2*, *Runx2, Tnfaip6*, *Ptx3*, *Areg*, *Ereg* in mGCs silencing *Adamts1*. **B, C** Quantitative RT-PCR (**B**) analysis and western blot (**C**) analysis of *Adamts1* and *Isg15* in ovaries from PMSG/hCG stimulated wild-type mice. **D** Western blot analysis of ADAMTS1 in mGCs transfected with 4 μg, 8 μg or 12 μg of FLAG-ISG15 plasmid, followed by cycloheximide (CHX) treatment for the indicated duration. **E** ISGylation level of ADAMTS1 in mGCs transfected with 4 μg, 8 μg or 12 μg of FLAG-ISG15 plasmid. Cell lysates were immunoprecipitated with anti-HA beads. **F** Western blot analysis of ADAMTS1 in mGCs treated with proteasome inhibitor or autophagy inhibitor. mGCs were transfected with HA-ADAMTS1, FLAG-ISG15, FLAG-UBA7, FLAG-UBE2L6, and FLAG-HERC6. Thirty-two hours after transfection, mGCs were treated with 20 μM MG132 (proteasome inhibitor) or 5 mM 3-MA (autophagy inhibitor) for 10 h. **G** Immunoprecipitation assays of ISGylation of ADAMTS1 in mGCs treated with MG132. mGCs were transfected with FLAG-ISG15, HA-ADAMTS1, FLAG-UBA7, FLAG-UBE2L6, FLAG-HERC6 and 20 μM MG132. Cell lysates were immunoprecipitated with anti-HA beads.** H** Western blot analysis of endogenous and exogenous ADAMTS1 protein in mGCs transfected with si-*Isg15*, HA-ADAMTS1 plasmid or not, and MG132. **I** ISGylation level of endogenous ADAMTS1 in mGCs transfected with si-*Isg15* and MG132.** J** ISGylation level of exogenous ADAMTS1 in mGCs transfected with si-*Isg15*, HA-ADAMTS1 plasmid and MG132. **K** Immunoprecipitation assays of ISGylated ADAMTS1 in mGCs treated with proteasomes. Purified ISGylated ADAMTS1 was incubated with 15 μM 20S or 26S proteasomes and analyzed by western blot. **L** Immunoprecipitation assays of ISGylation and ubiquitination in mGCs transfected with FLAG-USP18 plasmid and MG132

In previous studies, ADAMTS1 was identified as an ovulation regulator; in this study, we found that *Adamts1* knockdown suppressed the expression of cumulus expansion-linked and ovulation-related genes in mGCs, including *Ptgs2*, *Has2*, *Runx2*, *Tnfaip6*, *Ptx3*, *Areg*, and *Ereg* (Fig. 7A). Moreover, ADAMTS1 was the most highly expressed while *Isg15* was the lowest expressed in ovaries at 12 h after PMSG/hCG injection during the ovulation stage (Fig. 7B, C), suggesting that ISG15 might negatively regulate ADAMTS1 expression. ISGylation influences the steady-state level and degradation of its target proteins [23], therefore the protein level of ADAMTS1 was measured. *Isg15* overexpression caused a marked decrease in ADAMTS1 protein but a sharp increase in ISGylated ADAMTS1 in mGCs (Fig. 7D, E and Fig. 4B), indicating that ISGlyation may induce the degradation of ADAMTS1 protein.

Next, the roles of protein degradation pathways in ISGylated ADAMTS1 degradation were examined in mGCs. We found that MG132 (proteasome inhibitor) but not 3-MA (autophagy inhibitor) could rescue the degradation of ADAMTS1 protein (Fig. 7F). In contrast, the ISGylation of ADAMTS1 was markedly increased in mGCs treated with proteasome inhibitor MG132 (Fig. 7G). *Isg15* knockdown could enhance the protein level of exogenous and endogenous ADAMTS1 but suppressed the ISGylation of exogenous and endogenous ADAMTS1, and this effect could be eliminated with MG132 treatment (Fig. 7H–J). Subsequently, upon incubation of purified ISGylated ADAMTS1 with 20S proteasome in vitro, the ISGylation of ADAMTS1 almost disappeared (Fig. 7K). An individual protein can be either ISGylated or ubiquitinated at the same lysine, which regulates the activity and degradation of this protein [24]. To determine which kind of protein PTMs were involved in ADAMTS1 degradation, we measured the ubiquitination and ISGylation level of ADAMTS1 in mGCs treated with the proteasome inhibitor (MG-132) or deconjugating enzyme *Usp18* plasmid. We found that ISGylation rather than ubiquitination of ADAMTS1 increased in MG132 treated mGCs, but decreased in *Usp18* treated mGCs (Fig. 7L). These results demonstrate that ISG15 induces the proteasome-dependent degradation of ADAMTS1.

### The transcription factor IRF3 stimulates Isg15 expression in mGCs

To identify the regulatory elements of *Isg15* expression in mGCs, a series of truncated *Isg15* promoters were used to determine promoter activity. TRANSFAC analyses and luciferase assays revealed a potential regulatory interferon regulatory factor 3 (IRF3) binding site (− 182 bp to − 172 bp) in the *Isg15* promoter region (Additional file 5: Fig. S4A, B). Subsequently, site-directed mutagenesis exhibited that the IRF3-element mutant (pGL3-*Isg15*-207-Mut) reduced *Isg15* promoter activity, suggesting that IRF3 could bind to the site (− 182 bp to − 172 bp) in the *Isg15* promoter (Additional file 5: Fig. S4C). Chromatin immunoprecipitation (ChIP) and electrophoretic mobility shift assays (EMSA) analysis demonstrated that IRF3 specifically bound to the *Isg15* promoter region in vitro and in vivo (Additional file 5: Fig. S4D, E). Moreover, IRF3 promoted the expression of *Isg15* and inhibited ovulation*-*related genes in mGCs but not in *Isg15* knockdown mGCs (Additional file 5: Fig. S4F, G). Additionally, IRF3 accelerated ADAMTS1 ISGylation and further degradation, whereas this effect was abrogated by *Isg15* knockdown (Additional file 5: Fig. S4H, I). These results suggest that IRF3 enhances endogenous *Isg15* expression by specifically binding to its promoter region, and facilitates ADAMTS1 degradation to prevent ovulation.

## Discussion

In our previous study, we reported that the level of *Isg15* mRNA in pre-ovulatory ovarian follicles of high-yielding sows (Meishan sows) was lower than that in low-yielding sows (Large White sows) [25, 26]. Therefore, ISG15 might be a negative regulator of ovarian folliculogenesis. Here, we found that *Isg15*^*−/−*^ female mice are hyper-fertile. Moreover, *Isg15*^*−/−*^ females displayed a more sensitive response to gonadotropin along with an increased cumulus expansion and ovulation rate. These findings document that *Isg15* deletion could facilitate ovulation process and then improve female fertility in mice.

ISG15 is best known as a 17-kD ubiquitin-like protein with two ubiquitin-like domains that are 30% homologous to ubiquitin [27]. Similar to ubiquitin, its carboxy-terminal LRLRGG motif is necessary both for recognition by ISG15-conjugating enzymes (E1, E2, E3) and for covalent conjugation to lysine residues of substrates. ISG15 modification (ISGylation) of substrates could signal to another protein that interacts with the substrates or directly alter the function of substrates [28]. Initially, ISG15 has been reported to exert its immunomodulatory and antiviral functions mainly through its covalent conjugation (ISGylation) [29]. For instance, ISGylation of NS1 protein of influenza A virus (NS1A) inhibits viral replication by disrupting the interactions between NS1A and importin-α, thereby increasing resistance to viral infection [30]. In addition, it has been reported that environmental stress significantly increases the rate of embryo mortality in *Isg15* knockout female mice [15]. However, recent reports have revealed that *Isg15* knockout female mice yield litters of normal size [31, 32], which suggests the role of ISG15 in female fertility is unclear. In this study, the silencing of *Isg15* and ISG15-conjugating enzymes (E1, E2, E3) upregulated the expression of ovulation-related genes in mGCs. Additionally, we found that *Isg15*^*−/−*^ female mice are hyper-fertile, which is ascribed to the increased ovulation rate. Overall, these results indicate that ISG15 inhibits ovulation and female fertility.

Currently, more than 300 proteins have been identified as potential ISGylation targets using labeled ISG15 through a proteomics approach [33]. Several cellular proteins involved in antiviral and inflammatory activity, including eukaryotic translation initiation factor 2-alpha kinase 2 (EIF2AK2), retinoic acid-inducible gene I (RIG-I), and Janus kinase 1 (JAK1), have also been identified as ISGylation substrates [34]. However, few of these target proteins are associated with ovulation, as ISGylation has not been analyzed in ovaries or ovarian cells in previous studies. To explore the role of ISG15-mediated ISGylation in ovulation, we performed mass spectrometric analysis and identified 57 potential proteins interacting with ISG15 in mGCs. In addition to the previously known RNF213 [20], we also identified novel proteins as potential ISGylation substrates, namely ribosomal protein S3 (RPS3) and protease serine 23 (PRSS23). RPS3, a DNA repair endonuclease and ribosomal protein, induces apoptosis through caspase-dependent JNK activation [35]. PRSS23 has been reported as a novel ovarian protease, which plays an important role in the transition from pre-antral into antral follicles [36]. In this study, a disintegrin-like and metalloproteinase with thrombospondin type 1 motif (ADAMTS1) stands out as an ISG15 candidate target, given that its activity is induced in ovarian follicles by ovulatory hormones and is required for female fertility [37, 38]. Moreover, recent reports have indicated that ADAMTS1 promotes cumulus expansion and pre-ovulatory follicle formation [39, 40], which is consistent with the phenotypes of *Isg15* knockout mice. In the present study, the results of the immunoprecipitation assays are in line with the MS findings, confirming that ADAMTS1 is a novel target of ISGylation in mGCs, which adds to our understanding of ISGylation substrates in follicular development and ovulation.

Numerous proteins have been suggested to be ISGylated, but little is known about the functions of ISGylation on target proteins. Recently, several studies have suggested that protein ISGylation could lead to either gain or loss of function. For instance, ISGylation of IRF3 disrupts the interaction between peptidyl-prolyl cis/trans isomerase 1 (PIN1) and IRF3, resulting in sustained IRF3 activation and enhanced antiviral activity [41]. Furthermore, ISGylation of filamin B blocks its scaffold function, impairing the IFN-induced JNK signaling pathway. Here, ISGylation of ADAMTS1 was found to negatively regulate the expression of ovulation-related genes by promoting ADAMTS1 proteolysis, which demonstrates that ISG15 disturbs ADAMTS1 function. Proteolysis is critical for maintaining cellular homeostasis and regulating the signaling pathway by which misfolded or damaged proteins are degraded into peptides or amino acids [42]. Generally, there are two kinds of proteolysis: autophagy-mediated lysosomal proteolysis and ubiquitin-mediated proteasomal proteolysis. Indeed, the ubiquitin–proteasome system (UPS) is the major pathway for protein degradation in eukaryotic cells [43]. Ubiquitinated proteins are directly recognized by the 26S proteasome or recruited to the proteasomes by ubiquitin receptors, and then are degraded into small peptides and reusable ubiquitin [44]. Notably, many researchers have suggested that ISGylation as a ubiquitination-like protein modification promotes the degradation of target proteins via a 20S proteasomal pathway [24]. Consistently, we observed that ISG15 facilitates the degradation of ISGylated ADAMTS1 by 20S proteasome in this study. These findings provide plenty of evidences that ISG15 is essential for ovulation by altering the stability of ADAMTS1 protein in a proteasome-dependent pathway.

Specific transcription factors induced by the LH surge play a critical role in ovulation by regulating the transcription of ovulation-related genes. As a primary transcriptional regulator, IRF3 is involved in inflammation, antiviral defense, and energy metabolism [19, 45]. Several type I interferon genes and interferon-stimulated genes are the targets of transcription factor IRF3 [46]. For example, IRF3 regulates glucose metabolism and adaptive thermogenesis by driving the expression of *Isg15* [19]. Our analysis shows that IRF3 binds to the promotor of *Isg15* and promotes its expression in mGCs. IRF3 decreases the expression of ovulation-related genes in control mGCs, but not in *Isg15* knockdown mGCs. Moreover, IRF3 facilitates ADAMTS1 ISGylation and degradation. Taken together, IRF3 is a positive regulator of *Isg15* expression and thereby participating in ISG15-mediated ovulation suppression.

**Fig. 8:**
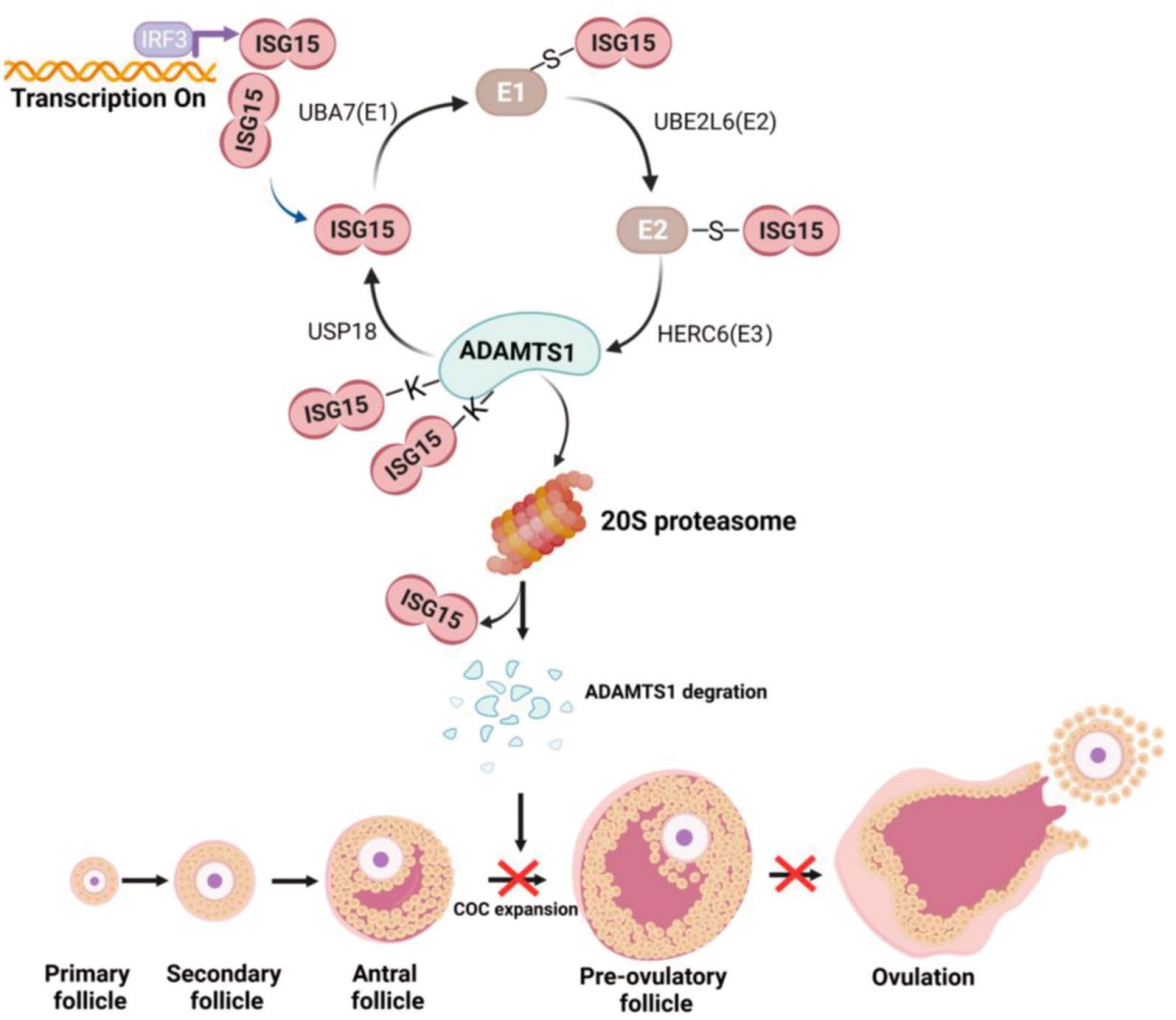
Schematic summary of the critical role of ISG15 in ovulation and female fertility through inducing ISGylation of ADAMTS1 in mice

In this study, we uncovered a previously unrecognized function of ISG15 in mammalian ovaries, and the results of our analyses indicate that ISG15 negatively regulates ovulation and female fertility. Indeed, *Isg15* knockout in mice could improve reproductive capacity with more sensitive ovarian responses to gonadotropin by promoting cumulus expansion and ovulation rate, which is due to prevent ISGylated ADAMTS1 degradation (Fig. 8). These findings contribute to a more complete understanding of the function of ISGylation in ovulation and indicate a potential approach to improve mammalian fertility.

## Conclusions

Based on the results of the present study, we conclude that ISG15 plays a pivotal role in cumulus expansion, and this role is dependent on ISG15-mediated ISGylation modification. The regulatory axis of ISG15-ADAMTS1 is required for ovulation and female fertility.

## Materials and methods

### Animal models

*Isg15*^−/−^ mice with the C57BL/6 background were generated using the CRISPR genome editing system. Two single guide RNAs (sgRNAs) targeting exon 2 of *Isg15* gene in mouse genome were designed using an online CRISPR design tool (http://tools.geneome-engineering.org), and inserted into the *px459* vector (48139, Addgene). PCR amplification was used for genotypic characterization of all mice. Primers used for genotyping are shown in Additional file 6: Table S2. All mice were housed in a pathogen-free environment with the temperature maintained at 20–22 °C and relative humidity at 50–70%, and were under a 12 h/12 h light/dark cycle. All mice had ad libitum accesses to standard chow diet.

All the animal procedures were approved by the Institutional Animal Care and Use Committee of Huazhong Agricultural University, and the mice were housed in the specific pathogen-free facility of Huazhong Agricultural University. All experiments with mice were conducted ethically according to the Guide for the Care and Use of Laboratory Animal guidelines.

### Superovulation and COC expansion assays

Female mice at 23 days of age were injected intraperitoneally with 5 IU pregnant mare serum gonadotropin (PMSG, B191009, Ningbo Sansheng Biotechnology) to stimulate preovulatory follicle development, and 48 h later injected with 5 IU human chorionic gonadotropin (hCG, B120207, Ningbo Sansheng Biotechnology) to stimulate ovulation. Ovaries were collected from these mice at 44 h after PMSG injection and 0 h, 8 h, 10 h, 16 h, 24 h after hCG injection, dissected and repeatedly punctured in M2 medium (M7167, Sigma). Cumulus-oocyte complexes (COCs) were released from antral follicles in *Isg15*^+/+^ and *Isg15*^−/−^ ovaries. To examine cumulus expansion, COCs were incubated for 0 h, 10 h, 16 h in serum-free Eagle's minimum essential medium (MEM, 11,095,080, Gibco) in the presence of 10 ng/ml epidermal growth factor (EGF, PHG0311L, Thermo Fisher Scientific) at 37 °C in a humidified atmosphere of 5% CO_2_. A subjective, 0 (no expansion) to 4 (full expansion) scoring system was used to evaluate the degree of cumulus expansion in mouse oocytes, as previously described [47]. After evaluation, oocytes were removed by 0.1% hyaluronidase (37326-33-3, Sigma) and cumulus cells were collected. In each independent experiment 50 COCs were scored.

### Natural ovulation and embryo count assay

For the natural ovulation assay, eight-week-old female mice were examined for the estrous cycle every day. Females in the estrus were mated with wild-type males. The next morning (at 8:00–9:00 AM) that the vaginal plug appeared in above females was designed as embryonic day (d 0.5). The oviducts were dissected from the females with vaginal plug, and zygotes were collected and counted. We called these collected zygotes as naturally ovulated oocytes. On the other hand, female mice were killed at early embryonic d 12 and the number of implanted embryos was counted.

### Fertility analyses and estrous cycle analyses

*Isg15*^+/+^ and *Isg15*^−/−^ females/males at eight weeks of age were mated with wild-type mice for a period of eight months, respectively. The number of litters and pups were recorded at the end of the fertility test. Vaginal smears were collected daily for 30 consecutive days by flushing the vaginal opening with 15 μl of 0.9% sterile saline and then transferred to glass slides to air dry. Dry smears were stained with 0.5% toluidine blue before examination under a light microscope. Estrous-cycle stage was determined based on the presence or absence of leukocytes, cornified epithelial cells, and nucleated epithelial cells [48].

### Histology hematoxylin–eosin (H&E) staining and immunofluorescence

Ovarian, testicular, and epididymal tissue samples from *Isg15*^+*/*+^ and *Isg15*^−/−^ mice were fixed in Bouin’s fixative, embedded in paraffin, and serially sectioned at 5 μm thickness. The tissue sections were then stained with hematoxylin–eosin (Sigma-Aldrich, USA), and follicles at various development stages were counted in every three ovarian sections based on morphological classification of follicles [49]. For immunofluorescence, tissue sections or granulosa cells were sequentially incubated with primary antibodies and secondary antibodies as described previously [49]. Digital images were acquired using an epifluorescence microscope (Olympus BX53) or confocal fluorescence microscopy (Zeiss LSM 800, Jena, Germany) with 4–100 × objectives. The fluorescence signals were quantitated using the NIH Image analysis program ImageJ. The antibodies used are shown in Additional file 7: Table S3.

### Assessment of sperm count and morphology

Sperms were isolated from cauda epididymis of eight-week-old *Isg15*^+*/*+^ and *Isg15*^−/−^ male mice. Then, sperms were incubated in 500 μl of TYH medium (M2050, Easycheck) for 30 min at 37 °C. The number of sperms was calculated using a cell counting plate. For sperm morphological analyses, cauda epididymal sperms were spread onto glass slides and stained with Giemsa (48900, Sigma).

### Cell culture and treatment

Granulosa cells from pre-ovulatory ovarian follicles of Kunming mice were isolated as described previously [50]. Murine primary granulosa cells were cultured in Dulbecco’s minimum essential medium DMEM/F12 (11320033, Gibco) supplemented with 10% fetal bovine serum (10099141C, Gibco), 100 U/ml penicillin (A3160802, Gibco) and 100 mg/ml streptomycin (15140122, Gibco) at 37 °C in a humidified atmosphere of 5% CO_2_. Cells were inoculated and grew up to 70% confluence at the time of treatment. For inhibitor treatment, mGCs were treated with 20 μM MG132 (proteasome inhibitor; 508338, Thermo Fisher Scientific) or 5 μM 3-MA (autophagy inhibitor, M9281, Sigma) at 10 h before harvesting the cells. For cycloheximide (CHX, 12320, MCE) treatment, mGCs were treated for 0 h, 2 h, 4 h, 8 h before harvesting the cells.

### SiRNA and plasmid transfection

Small interfering RNAs (siRNAs) targeting *Isg15*, *Uba7*, *Ube2l6*, *Herc6, Trim25*, *Usp18* and *Irf3* were synthesized by GenePharma (Suzhou, China). The full-length coding sequence (CDS) of *Isg15*, *Uba7*, *Ube2l6*, *Trim25*, *Herc6, Trim25*, *Usp18, Adamts1*, *Irf3* genes, site-directed mutants and deletion fragments of *Isg15*, *Adamts1*, *Herc6* and *Irf3* genes were obtained using PCR. Above fragments were separately cloned into *pcDNA3.1*( +) vector (D2951, Beyotim), pCMV-FLAG (D2632, Beyotime), pCMV-HA (D2639, Beyotime), or pGL3-basic vector (E1751, Promega). Plasmids and siRNAs were transfected into mGCs using Lipofectamine™ 3000 (L3000015, Invitrogen™) and RNAiMAX transfection reagent (13778030, Invitrogen™), respectively.

### Reproductive hormone measurement

For measuring serum hormone, mice were anesthetized and blood was collected by cardiac puncture. Serum hormone levels were determined by follicle stimulating hormone ELISA kit (KA2330, Novus Biologicals), luteinizing hormone ELISA kit (KA2332, Novus Biologicals), and testosterone ELISA kit (RK00724, ABclonal), respectively.

### Luciferase reporter assays

Each plasmid was transfected with 500 ng recombinant constructs, together with 50 ng per well of pRL-TK (E2241, Promega). After transfection for 24 h, cells were collected and luciferase activities were measured using the dual-luciferase reporter assay system (E1910, Promega) according to the manufacturer’s instructions.

### Quantitative real-time PCR (qRT-PCR) analysis

Total RNA was extracted using the TRIzol reagent (15596026, Thermo Fisher Scientific), and treated with RNase-free DNase (M610A, Promega). Total RNA was reverse transcribed to cDNA using RevertAid RT reverse transcription kit (K1691, Thermo Fisher Scientific). Quantitative real-time PCR was performed using the iTaq™ universal SYBR green supermix (172–5121, Bio-Rad) and analyzed using CFX384 Touch™ real-time PCR detection system (Bio-Rad, USA). Primers used in the qRT-PCR are shown in Additional file 6: Table S2. Gene expression levels were normalized to the expression of *β-actin.* Relative expression levels were calculated using 2^−ΔΔCt^ method [51].

### Western blot analysis

Western blot was performed following the standard procedures [52]. Briefly, protein lysates were generated using RIPA lysis buffer (P0013B, Beyotime) with 1% protease inhibitor cocktail (HY-K0010T, MCE), 1% phosphatase inhibitor cocktail I (HY-K0021, MCE) and 1% phosphatase inhibitor cocktail II (HY-K0022, MCE). Protein extracts were separated in 10% SDS–polyacrylamide gels and then transferred onto 0.22 μm PVDF membranes (iseq00010, Millipore). The membranes were blocked with 5% non-fat dried milk or 3% BSA in TBST (20 mM Tris–HCl, pH 7.5, 150 mM NaCl, 0.1% Tween-20), incubated with primary antibody overnight at 4 °C, washed with 0.05% Tween in PBS three times, and incubated with HRP-coupled secondary antibody for 2 h at 37 °C. The ECL substrate kit (170–5061, Bio-Rad) was used to detect immunoreactive protein bands imaged by ChemiDocMP imaging system (Bio-Rad, USA). ImageJ was used to quantify the signal. The antibodies used are shown in Additional file 7: Table S3.

### Immunoprecipitation and mass spectrometry (IP-MS)

Cell lysates were incubated with anti-FLAG magnetic beads (B26101, Bimake) or anti-HA magnetic beads (B26201, Bimake) overnight at 4 °C. The beads were collected by centrifugation and then washed with PBS containing 0.1% Tween-20 (PBST). Bound proteins were eluted with loading buffer (50 mM Tris–HCl, 2% SDS, 1% mercaptoethanol, 10% glycerol, 0.1% bromophenol blue, pH 6.8), separated by SDS-PAGE and immunoblotted with appropriate antibodies. Protein digestion and mass spectrometry analyses were performed by Novogene Company (Novogene, Beijing, China).

### Chromatin immunoprecipitation (ChIP)

The ChIP experiment was carried on using the EZ-Magna ChIP™ A/G kit (17–371, Millipore). mGCs were fixed in 1% formaldehyde and quenched by 0.125 M glycine. Then the isolated chromatin DNA was sonicated and sheared to yield the fragments of 200–800 bp in length with the AVCX130 system (Sonics & Materials, Newtown, CT, USA). The sheared chromatin was immunoprecipitated with anti-HA or anti-IgG antibodies, respectively. The co-precipitated DNA was purified by DNA purification kit (D0033, Beyotime) and then subjected to qPCR. The details of specific primers for ChIP-qPCR are listed in Additional file 6: Table S2.

### Electrophoretic mobility shift assay (EMSA)

EMSA experiment was carried out using the LightShift chemiluminescent EMSA kit (20,148, Thermo Fisher Scientific). The nuclear extracts were isolated with a nucleoprotein extraction kit (P0028, Beyotime). Nuclear extract proteins (5.0 μg) were incubated in 10 μl reaction volume containing 4% glycerol, 1 mM MgCl_2_, 0.5 mM dithiothreitol, 0.5 mM EDTA, 50 mM NaCl, 10 mM Tris–HCl, pH 7.5 and 2.0 μg poly (dI-dC) with or without 50-fold excess of unlabeled DNA competitors on ice for 10 min followed by the addition of the biotin-labeled probes. For supershift assays, anti-HA antibody was added to the reaction mixture 20 min before the addition of the probes. All DNA–protein complexes were resolved by electrophoresis on 10% native polyacrylamide gel.

### In silico* sequence analysis*

Neural network promoter prediction software (http://www.fruitfly.org/seq_tools/promoter.html) was used to analyze the potential promoter region of *Isg15* gene. TRANSFAC^®^ Public 6.0. (http://gene-regulation.com/pub/programs.html ) was used to predict the transcription factor binding sites. The schematic summary was created with BioRender.com.

### Statistical analysis

Experiments were performed at least three times and data are expressed as mean ± SD. Statistical differences were determined by two-tailed Student’s t-test using Prism or Microsoft Excel. P < 0.05 was considered statistically significant.

## Supplementary Information

**Additional file 1: Figure S1.** Isg15 knockout does not alter male fertility.Quantitative RT-PCRanalysis and western blotanalysis of ISG15 in testes of three-week-old Isg15+/+ and Isg15−/− males. The right bar graph showed the quantification of protein level.Immunofluorescence analysis of ISG15 in testes of three-week-old Isg15+/+ and Isg15−/− males. Red: ISG15; Blue: DAPI; Scale bar: 200 μm.Cumulating number of pups born per Isg15+/+and Isg15−/−male mice when bred with Isg15+/+ female mice during the eight-month period of fertility test.Assessment of serum hormone levels in three-week-oldand eight-week-oldIsg15+/+ and Isg15−/− males.The representative testis morphology of three-week-old and eight-week-old Isg15+/+ and Isg15−/− males. The right is the ratio of testis weight to body weight.H&E staining of testis and epididymis sections in three-week-oldand eight-week-oldIsg15+/+ and Isg15−/− males. Scale bar: 100 μm.Sperm countof eight-week-old Isg15+/+ and Isg15−/− males.Rate of abnormal sperms in eight-week-old Isg15+/+ and Isg15−/− males. All data are mean ± SD; ***P < 0.001.

**Additional file 2: Figure S2.** Isg15 deficiency enhances the expression of cumulus expansion-linked genes in mouse ovaries.Immunofluorescence analysis of ADAMTS1, PTGS2, and PTX3in ovaries of three-week-old Isg15+/+ and Isg15−/− mice that were stimulated by PMSG/hCG. Scale bar: 50 μm.

**Additional file 3: Figure S3.** Knockdown of Isg15 and ISGylation system genes enhances the expression of ovulation-related genes in mGCs.Quantitative RT-PCRanalysis and western blotanalysis of ovulation-related genes including Adamts1, Ptgs2, Has2, Areg, Ereg in mGCs silencing Isg15.ISGylation level in Isg15+/+ and Isg15−/− mice ovaries.Quantitative RT-PCRanalysis and western blotanalysis of ovulation-related genes including Adamts1, Ptgs2, Has2, Areg, Ereg, Isg15 in mGCs silencing E1 activating enzyme Uba7.Quantitative RT-PCRanalysis and western blotanalysis of ovulation-related genes including Adamts1, Ptgs2, Has2, Areg, Ereg, Isg15 in mGCs silencing E2 conjugating enzyme Ube2l6.Quantitative RT-PCRanalysis and western blotanalysis of ovulation-related genes including Adamts1, Ptgs2, Has2, Areg, Ereg, Isg15 in mGCs silencing E3 ligating enzyme Herc6.Quantitative RT-PCRanalysis and western blotanalysis of ovulation-related genes including Adamts1, Ptgs2, Has2, Areg, Ereg, Isg15 in mGCs silencing E3 ligating enzyme Trim25.Quantitative RT-PCRanalysis and western blotanalysis of ovulation-related genes including Adamts1, Ptgs2, Has2, Areg, Ereg, Isg15 in mGCs silencing ISGylation-specific deconjugating enzyme Usp18. All data are mean ± SD; *P < 0.05, **P < 0.01.

**Additional file 4: Table S1.** Identification of ISG15 interacting partners in mGCs.

**Additional file 5: Figure S4.** IRF3 negatively regulates ovulation-related genes expression through ISG15-ADAMTS1 axis.Luciferase activity assays of a series of truncated Isg15 promoters in mGCs. Each truncated fragment linked with the luciferase gene in the pGL3-basic vector is shown in the left panel. The relative activities of these constructs determined by luciferase assays are shown in the right panel. The pGL3-basic vector was used as a negative control.Sequence analysis of IRF3 binding site of Isg15 promoter in mouse.Luciferase activity assays of the Isg15 wild-type and mutant promoter in mGCs. IRF3 binding site is indicated by empty box. The filled box shows the corresponding mutation. The pGL3-basic vector was used as a negative control.ChIP assays of IRF3 binding site in Isg15 promoter region. mGCs were transfected with HA-IRF3. The DNA fragments interacting with IRF3 protein were pulled down by anti-HA antibody and analyzed by qPCR. Total chromatin was used as the input. IgG was used as a negative control.EMSA assays of IRF3 binding site in Isg15 promoter region. Nuclear extracts were incubated with biotin-labeled probes in the absence or presence of various unlabeled probes and anti-HA antibody. Free probes are at the bottom of the gel, and the specific DNA-protein complex and DNA-protein-antibody complex bands are indicated by arrows. The sequences of the various probes are shown under the panel.Quantitative RT-PCRanalysis and western blotanalysis of ovulation-related genes including Adamts1, Ptgs2, Has2, Areg, Ereg, Isg15 in mGCs silencing Isg15 and overexpressing Irf3.Immunoprecipitation assays of ISGylated ADAMTS1 in mGCs silencing Isg15 and overexpressing Irf3.Western blot analysis of ADAMTS1 in mGCs silencing Isg15 and overexpressing Irf3. All data are mean ± SD; *P < 0.05, **P < 0.01.

**Additional file 6: Table S2.** The sequences of primers and siRNAs used in this study.

**Additional file 7: Table S3.** The details of antibodies used in the study.

## Data Availability

The datasets used and analyzed during the current study are available from the corresponding author on reasonable request.

## Abbreviations

ADAMTS1: A disintegrin-like and metalloproteinase with thrombospondin type 1 motif
ChIP: Chromatin immunoprecipitation
COCs: Cumulus-oocyte complexes
EIF2AK2: Eukaryotic translation initiation factor 2-alpha kinase 2
EMSA: Electrophoretic mobility shift assay
FSH: Follicle-stimulating hormone
H&E: Hematoxylin and eosin
HERC6: Hect domain and RLD 6
IFN: Interferon
IP-MS: Immunoprecipitation mass spectrometry
IRF3: Interferon regulatory factor 3
ISG15: Interferon-stimulated gene 15
JAK-1: Janus kinase 1
LH: Luteinizing hormone
mGC: Murine granulosa cell
NS1A: NS1 protein of the influenza A virus
PIN1: Peptidyl-prolyl cis/trans isomerase 1
PMSG/hCG: Pregnant mare serum gonadotropin/human chorionic gonadotropin
PP2A-A: Protein phosphatase 2A scaffold subunit
PTM: Post-translational modification
RIG-1: Retinoic acid-inducible gene I
RNF213: Ring finger protein 213
T: Testosterone
TRIM25: Tripartite motif-containing 25
UBA7: Ubiquitin-like modifier activating enzyme 7
UBE2L6: Ubiquitin-conjugating enzyme E2 L6
USP18: Ubiquitin carboxy-terminal hydrolase 18
