# Supplementary figures and images for: ISG15 suppresses ovulation and female fertility by ISGylating ADAMTS1
Source: Cell Biosci. 2023 May 11;13:84. doi: 10.1186/s13578-023-01024-4 (PMC10176748; doi:10.1186/s13578-023-01024-4)

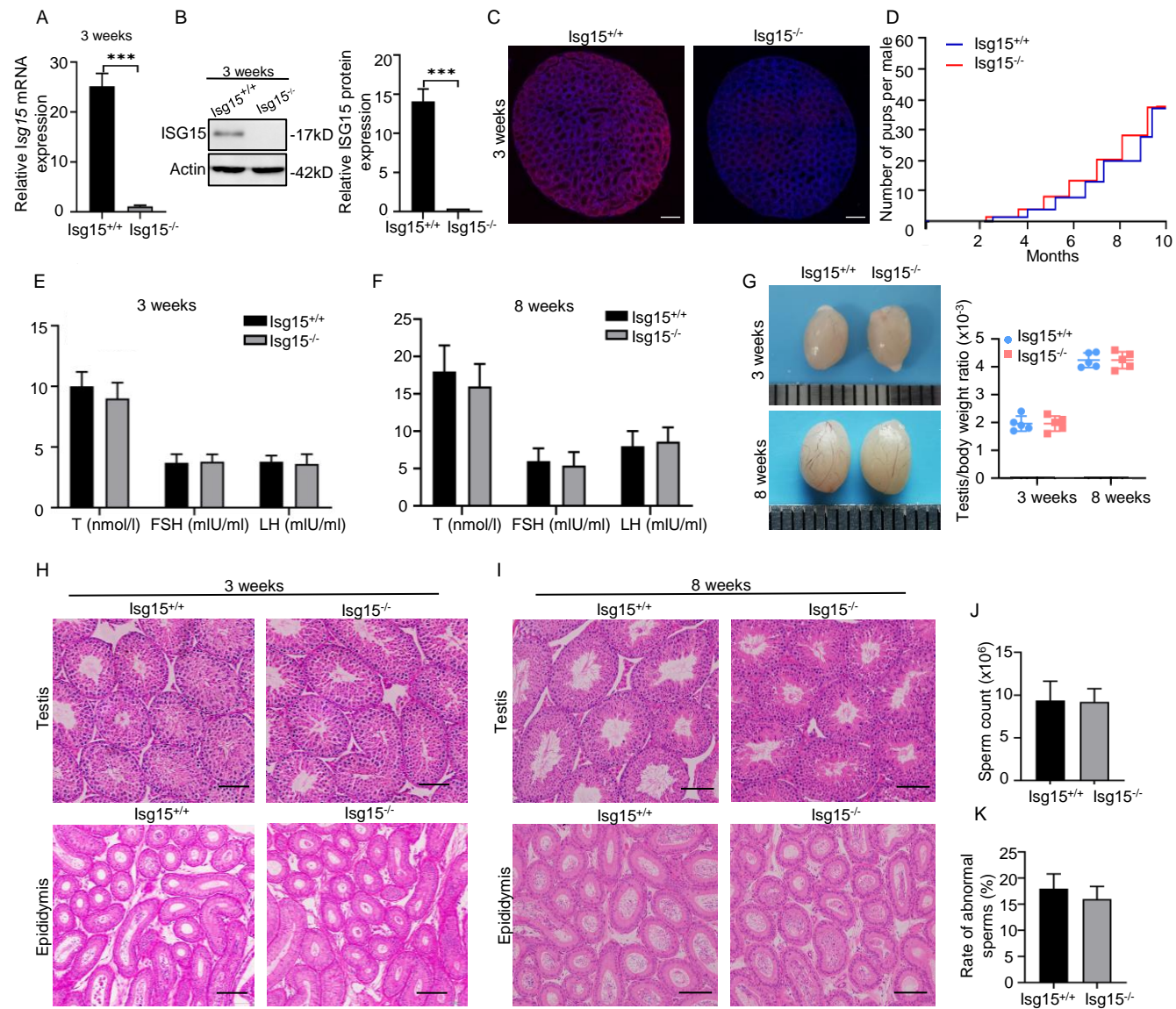

Supplement: Supplementary file 1 — Additional file 1: Figure S1. Isg15 knockout does not alter male fertility.Quantitative RT-PCRanalysis and western blotanalysis of ISG15 in testes of three-week-old Isg15+/+ and Isg15−/− males. The right bar graph showed the quantification of protein level.Immunofluorescence analysis of ISG15 in testes of three-week-old Isg15+/+ and Isg15−/− males. Red: ISG15; Blue: DAPI; Scale bar: 200 μm.Cumulating number of pups born per Isg15+/+and Isg15−/−male mice when bred with Isg15+/+ female mice during the eight-month period of fertility test.Assessment of serum hormone levels in three-week-oldand eight-week-oldIsg15+/+ and Isg15−/− males.The representative testis morphology of three-week-old and eight-week-old Isg15+/+ and Isg15−/− males. The right is the ratio of testis weight to body weight.H&E staining of testis and epididymis sections in three-week-oldand eight-week-oldIsg15+/+ and Isg15−/− males. Scale bar: 100 μm.Sperm countof eight-week-old Isg15+/+ and Isg15−/− males.Rate of abnormal sperms in eight-week-old Isg15+/+ and Isg15−/− males. All data are mean ± SD; ***P < 0.001. [file 13578_2023_1024_MOESM1_ESM.pdf]

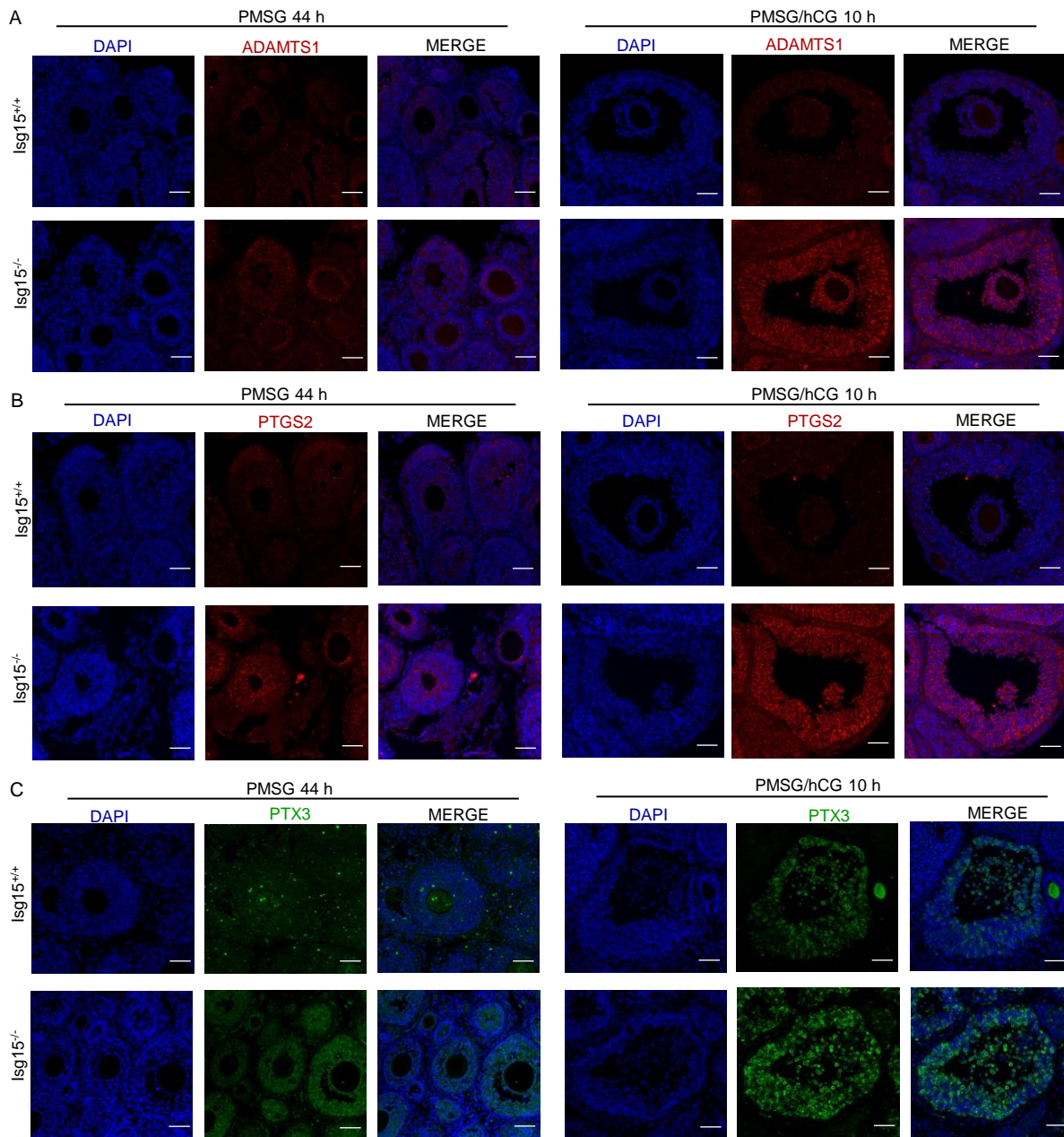

Supplement: Supplementary file 2 — Additional file 2: Figure S2. Isg15 deficiency enhances the expression of cumulus expansion-linked genes in mouse ovaries.Immunofluorescence analysis of ADAMTS1, PTGS2, and PTX3in ovaries of three-week-old Isg15+/+ and Isg15−/− mice that were stimulated by PMSG/hCG. Scale bar: 50 μm. [file 13578_2023_1024_MOESM2_ESM.pdf]

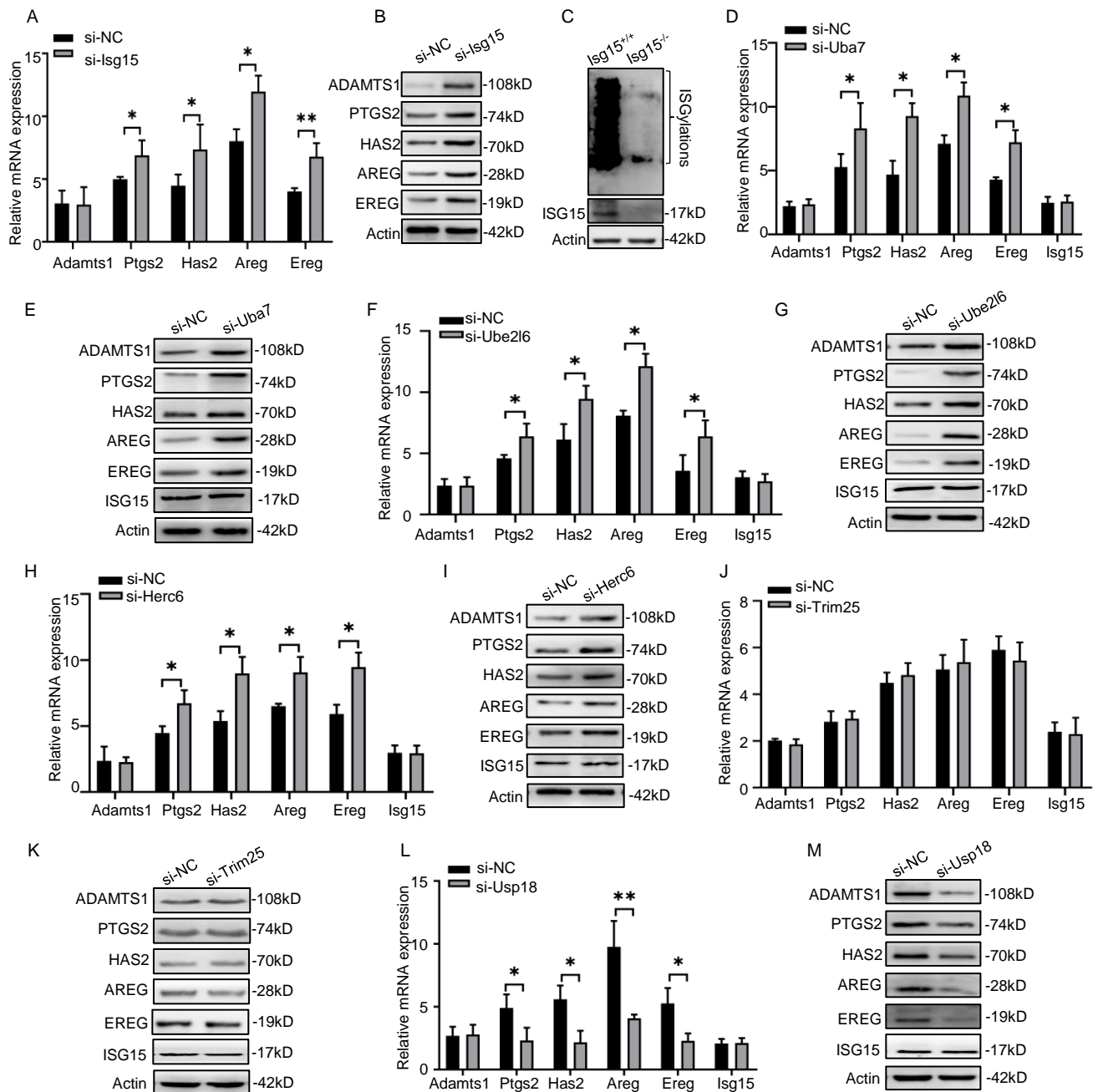

Supplement: Supplementary file 3 — Additional file 3: Figure S3. Knockdown of Isg15 and ISGylation system genes enhances the expression of ovulation-related genes in mGCs.Quantitative RT-PCRanalysis and western blotanalysis of ovulation-related genes including Adamts1, Ptgs2, Has2, Areg, Ereg in mGCs silencing Isg15.ISGylation level in Isg15+/+ and Isg15−/− mice ovaries.Quantitative RT-PCRanalysis and western blotanalysis of ovulation-related genes including Adamts1, Ptgs2, Has2, Areg, Ereg, Isg15 in mGCs silencing E1 activating enzyme Uba7.Quantitative RT-PCRanalysis and western blotanalysis of ovulation-related genes including Adamts1, Ptgs2, Has2, Areg, Ereg, Isg15 in mGCs silencing E2 conjugating enzyme Ube2l6.Quantitative RT-PCRanalysis and western blotanalysis of ovulation-related genes including Adamts1, Ptgs2, Has2, Areg, Ereg, Isg15 in mGCs silencing E3 ligating enzyme Herc6.Quantitative RT-PCRanalysis and western blotanalysis of ovulation-related genes including Adamts1, Ptgs2, Has2, Areg, Ereg, Isg15 in mGCs silencing E3 ligating enzyme Trim25.Quantitative RT-PCRanalysis and western blotanalysis of ovulation-related genes including Adamts1, Ptgs2, Has2, Areg, Ereg, Isg15 in mGCs silencing ISGylation-specific deconjugating enzyme Usp18. All data are mean ± SD; *P < 0.05, **P < 0.01. [file 13578_2023_1024_MOESM3_ESM.pdf]

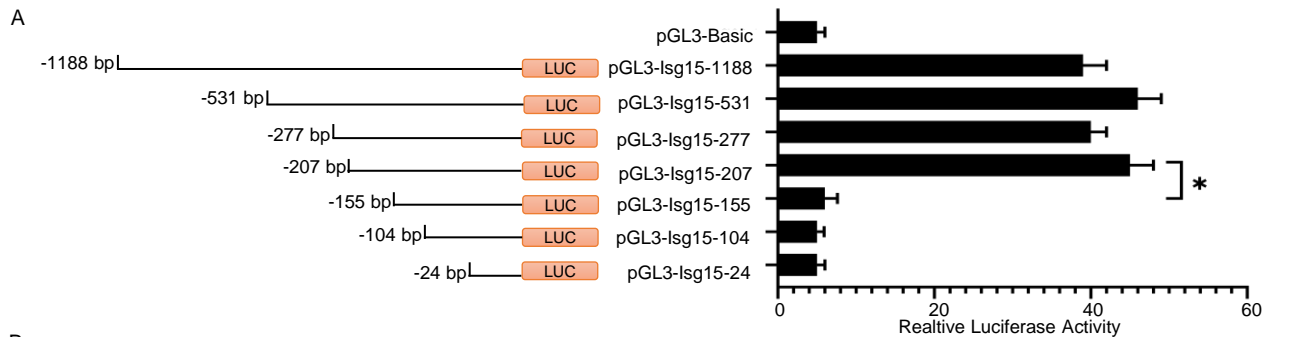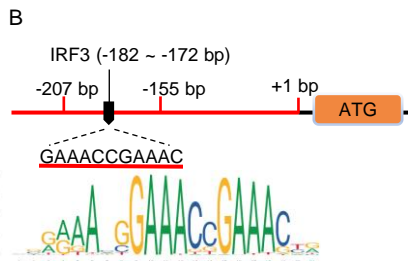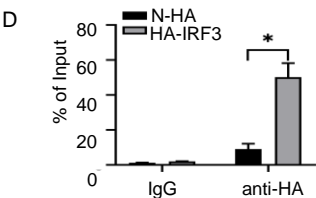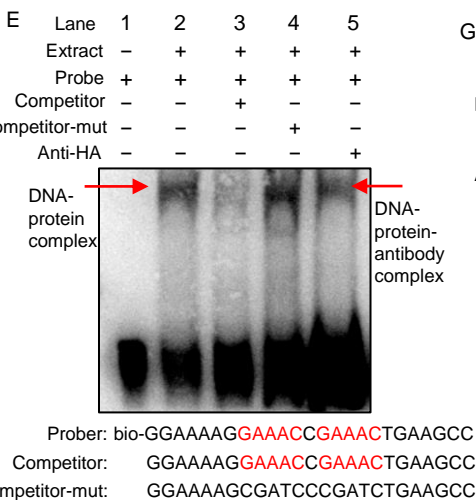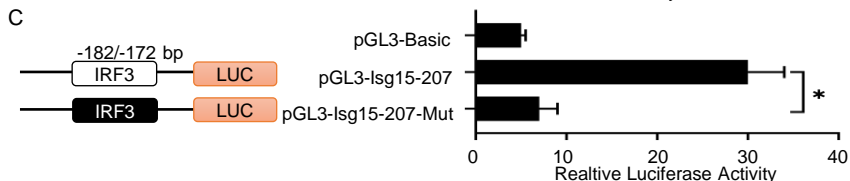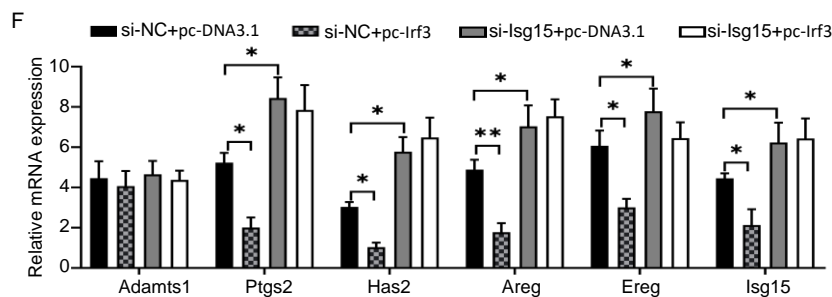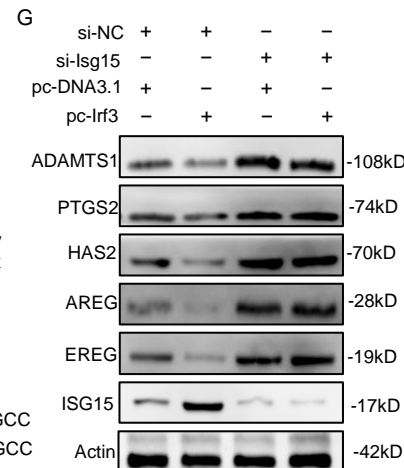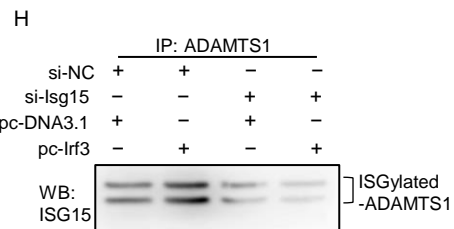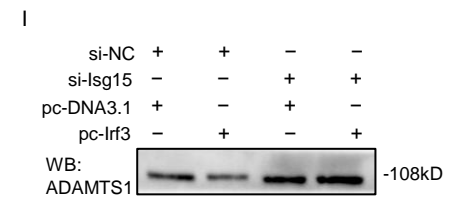

Supplement: Supplementary file 5 — Additional file 5: Figure S4. IRF3 negatively regulates ovulation-related genes expression through ISG15-ADAMTS1 axis.Luciferase activity assays of a series of truncated Isg15 promoters in mGCs. Each truncated fragment linked with the luciferase gene in the pGL3-basic vector is shown in the left panel. The relative activities of these constructs determined by luciferase assays are shown in the right panel. The pGL3-basic vector was used as a negative control.Sequence analysis of IRF3 binding site of Isg15 promoter in mouse.Luciferase activity assays of the Isg15 wild-type and mutant promoter in mGCs. IRF3 binding site is indicated by empty box. The filled box shows the corresponding mutation. The pGL3-basic vector was used as a negative control.ChIP assays of IRF3 binding site in Isg15 promoter region. mGCs were transfected with HA-IRF3. The DNA fragments interacting with IRF3 protein were pulled down by anti-HA antibody and analyzed by qPCR. Total chromatin was used as the input. IgG was used as a negative control.EMSA assays of IRF3 binding site in Isg15 promoter region. Nuclear extracts were incubated with biotin-labeled probes in the absence or presence of various unlabeled probes and anti-HA antibody. Free probes are at the bottom of the gel, and the specific DNA-protein complex and DNA-protein-antibody complex bands are indicated by arrows. The sequences of the various probes are shown under the panel.Quantitative RT-PCRanalysis and western blotanalysis of ovulation-related genes including Adamts1, Ptgs2, Has2, Areg, Ereg, Isg15 in mGCs silencing Isg15 and overexpressing Irf3.Immunoprecipitation assays of ISGylated ADAMTS1 in mGCs silencing Isg15 and overexpressing Irf3.Western blot analysis of ADAMTS1 in mGCs silencing Isg15 and overexpressing Irf3. All data are mean ± SD; *P < 0.05, **P < 0.01. [file 13578_2023_1024_MOESM5_ESM.pdf]
